# Routine Surveillance of SARS-CoV-2 Serostatus in Pediatrics Allows Monitoring of Humoral Response

**DOI:** 10.3390/microorganisms11122919

**Published:** 2023-12-04

**Authors:** Felix Wachter, Ferdinand Knieling, Roman Raming, David Simon, Joachim Woelfle, André Hoerning, Antje Neubert, Manfred Rauh, Adrian P. Regensburger

**Affiliations:** 1 Department of Pediatrics and Adolescent Medicine, Friedrich-Alexander-University (FAU) Erlangen-Nürnberg, 91054 Erlangen, Germany; felix.wachter@web.de (F.W.); roman.raming@uk-erlangen.de (R.R.);; 2 Department of Internal Medicine 3, Friedrich-Alexander-University (FAU) Erlangen-Nürnberg, 91054 Erlangen, Germany; david.simon@uk-erlangen.de

**Keywords:** COVID-19, pediatrics, seroprevalence, SARS-CoV-2, Omicron, surveillance

## Abstract

The occurrence of SARS-CoV-2 infections during the pandemic was mainly based on PCR testing of symptomatic patients. However, with new variants, vaccinations, and the changing of the clinical disease severity, knowledge about general immunity is elusive. For public health systems, timely knowledge of these conditions is essential, but it is particularly scarce for the pediatric population. Therefore, in this study, we wanted to investigate the spike and nucleocapsid seroprevalence in pediatric patients using routine residual blood tests collected during the pandemic. This prospective observational study was conducted over seven one-month periods. Herein, the latest four time periods (November 2021, January 2022, March 2022, and May 2022) are depicted. Each patient of a tertiary-care center in Germany was anonymized after collection of clinical diagnosis (ICD-10) and then routinely tested for the respective spike and nucleocapsid SARS-CoV-2 antibody titer. A total of 3235 blood samples from four time periods were included. Spike seroprevalence rose from 37.6% to 51.9% to 70.5% to 85.1% and nucleocapsid seroprevalence from 11.6% to 17.0% to 36.7% to 58.1% in May 2022. In detail, significant changes in seroprevalence between age groups but not between sex or diagnosis groups were found. Quantitative measures revealed rising spike and constant nucleocapsid antibody levels over the pandemic with a half-life of 102 days for spike and 45 days for nucleocapsid antibodies. Routine laboratory assessment of SARS-CoV-2 in residual blood specimens of pediatric hospitals enables monitoring of the seroprevalence and may allow inferences about general immunity in this cohort.

## 1. Introduction

Three years after the onset of the SARS-CoV-2 pandemic, COVID-19 continues to present significant challenges to health care providers and political decision makers on a global scale [1]. Current attention has shifted towards long-term ramifications, including post-COVID-19 sequalae. A notable quarter of children exhibit postacute symptoms following SARS-CoV-2 infection [2], and there is a growing body of evidence indicating persistent pulmonary dysfunction in some children with post-COVID-19, even months after the initial infection [3]. The scale of affected individuals may be considerable, with the World Health Organization reporting over 750 million confirmed infections worldwide and nearly 7 million deaths [4]. Seroprevalence data further suggest that the actual numbers could be considerably higher, particularly among the pediatric population [5,6,7]. Through the utilization of routine laboratory blood samples for SARS-CoV-2 prevalence monitoring, we previously demonstrated that the true number of infections may be 3.93–5.66 times higher [8]. This is consistent with the findings of a similar nationwide study involving adults [9]. While current surveillance strategies predominantly rely on PCR testing, these revelations imply a significant underestimation by this method. Seroprevalence studies have the potential to yield more accurate results, revealing the genuine extent of infections. Consequently, they represent an indispensable tool for comprehending the dynamics of the pandemic and estimating the proportion of immune individuals within the population. Although the COVID-19 pandemic evolved to endemic since it began [10], continuing to monitor infection events is important, as irregular wave patterns with low predictability of infection rates are expected [11]. Furthermore, coincidence with other infections, such as seasonal and endemic influenza waves, has to be expected. Unfortunately, co-infection of both SARS-CoV-2 and influenza aggravates clinical symptoms in children [12].

However, one substantial challenge associated with seroprevalence studies is the considerable allocation of resources, both in terms of finances and workforce. Considerable staffing is required to secure consent and obtain patient samples, incurring substantial expenses. As the pandemic endures, there is a growing need to establish cost-effective methodologies for ongoing seroprevalence monitoring. Studies involving extensive recruitment procedures and labor-intensive patient sample collection may become infeasible in the long term. Therefore, there is a compelling need for innovative study protocols. It is noteworthy that many of the published seroprevalence studies excluded children. This may be attributed to the complexities in obtaining the requisite sample material, which is particularly challenging in this age group due to ethical considerations surrounding blood collection from children for academic purposes with no direct individual benefit. Consequently, there is a paucity of current pediatric serological data, which are vital for comprehending the intricate interplay between multiple infections and vaccinations among children during the course of the pandemic. Despite a supposed vaccination rate of 22.4% among children under 12 years and 74.5% among those aged 12–17 years in Germany [13], the actual rate remains uncertain. Moreover, detailed insights into antibody kinetics to facilitate interpretation are limited [14,15,16]. Once again, this gap in knowledge is most pronounced in the pediatric population due to the aforementioned challenges. This is a significant issue as understanding antibody kinetics, especially antibody half-life, is crucial for interpreting seroprevalence study findings as well as assessing the duration of humoral immunity following infection and vaccination. It is also instrumental in interpreting antibody assay results within a clinical diagnostic context. Consequently, we undertook a prospective observational study to assess SARS-CoV-2 serostatus with the aim of monitoring seroprevalence, the humoral immune response, and antibody dynamics in the pediatric population.

## 2. Materials and Methods

### 2.1. Study Design

In this repeated cross-sectional study, we included residual blood samples from pediatric patients treated at the University Hospital of the Friedrich-Alexander University Erlangen-Nürnberg as described previously [8]. In addition, we evaluated four consecutive time periods: November 2021, January 2022 (including 1 February 2022), March 2022, and May 2022. Individual consent was not required, as all tests were performed anonymously. Specimen inclusion criteria were as follows: residual samples (lithium heparin plasma) of every outpatient as well as inpatients under 18 years of age that contained enough residual material after diagnostics were included. Therefore, only blood samples that were drawn for other indications were used. No additional patient material was obtained for the purpose of this study. Exclusion criteria were insufficient material or age above 18 years. All samples were frozen and stored in the clinical routine laboratory. After collection of clinical data, the samples were anonymized and then analyzed in batches for SARS-CoV-2 antibodies using two assays, one targeting the spike and the other one targeting the nucleocapsid antigen. 

### 2.2. Antibody Procedures and Analyses

An Elecsys^®^ (Roche Diagnostics, Mannheim, Germany) Anti-SARS-CoV-2 S antibody assay to detect spike antibodies and an Elecsys^®^ Anti-SARS-CoV-2 antibody assay to detect nucleocapsid antibodies were used according to the manufacturer’s protocol and as described previously [8]. All tests were performed in the clinical routine laboratory of the Department of Pediatrics and Adolescent Medicine, Friedrich-Alexander University (FAU) Erlangen-Nürnberg. In brief, the samples were stored for a week to allow for any potential additional testing that might be required for the patients before analyzing SARS-CoV-2 antibodies. Subsequently, the samples were processed in batches. Test results exceeding 0.8 U/mL were classified as reactive based on the manufacturer’s recommended cutoff. The assay’s linear range spans values between 0.40 and 250 U/mL. For samples with values above 250 U/mL, an automatic dilution process was initiated using Diluent Universal (Roche Diagnostics, Mannheim, Germany). In this study, the realized dilutions were either 1:10 or 1:100. The analyzer then calculated results by multiplying them with the appropriate dilution factor, effectively enabling an upper limit of quantification of 25,000 U/mL for these analyses. Only four samples did not require dilution beyond 250 U/mL or 2500 U/mL. The assigned U/mL values are equivalent to binding antibody units (BAU)/mL as defined by the World Health Organization (WHO) in their first International Standard for anti-SARS-CoV-2 immunoglobulin (NIBSC code 20/136).

For each sample period, every participant was only included once for seroprevalence analyses. In patients with multiple blood samples, the latest was used for analyses only. Sex and age were profiled through descriptive statistics, where sex was presented as the count and percentage, while age was represented by the median and interquartile range (IQR). Seroprevalence of anti-S/anti-N for each time period was determined by dividing the count of patients with anti-S/anti-N reactive samples by the total number of included samples. For the calculation of antibody half-time, every serial sample with a minimum time interval of 10 days was included. Half-life was then calculated using T1/2 = LN(2)/LN(sample 2/sample 1) multiplied by the date difference between the two samples. For the correlation of spike and nucleocapsid antibody levels, the samples of newborns were excluded, as they represent the immunity of their mothers. 

### 2.3. Clinical Data

Demographic data were collected including sex and age of each participant. Ages were subdivided into groups as labeled. The group of newborns was defined as participants of 28 days and younger. Patients were further categorized according to the ICD-10 code and abbreviated as follows: congenital malformations; deformations and chromosomal abnormalities: deformities; certain infectious and parasitic diseases: infectiology; certain conditions originating in the perinatal period: neonatology, endocrine, nutritional, and metabolic diseases: endocrinology; factors influencing health status and contact with health services: health services; diseases of the skin and subcutaneous tissue: dermatology, diseases of the respiratory system: pulmonology; diseases of the eye and adnexa and diseases of the ear and mastoid process: ear and eye; diseases of the blood and blood-forming organs and certain disorders involving the immune mechanism: hematology; diseases of the circulatory system: cardiology; diseases of the musculoskeletal system and connective tissue: muscle disease; diseases of the nervous system: neurology; diseases of the genitourinary system: urology; diseases of the digestive system: gastroenterology; neoplasms: neoplasms; mental and behavioral disorders: psychiatry, symptoms, signs, and abnormal clinical and laboratory findings; not elsewhere classified: symptoms and lab results; injury, poisoning, and certain other consequences of external causes: injury and poisoning.

### 2.4. Statistics

Statistical analyses were performed using GraphPad Prism (GraphPad software LCC, version 10.0.0, Boston, MA, USA). Seroprevalence status was compared between time periods for all samples, within time periods between male and female samples, and for each age group and the rest of the cohort by Fisher’s exact test. Antibody levels were compared by Kruskal–Wallis test. For correlation analyses, Spearman (rs) coefficients are given. *p* values < 0.05 indicate statistical significance. 

## 3. Results

### 3.1. Participants

**Figure 1:**
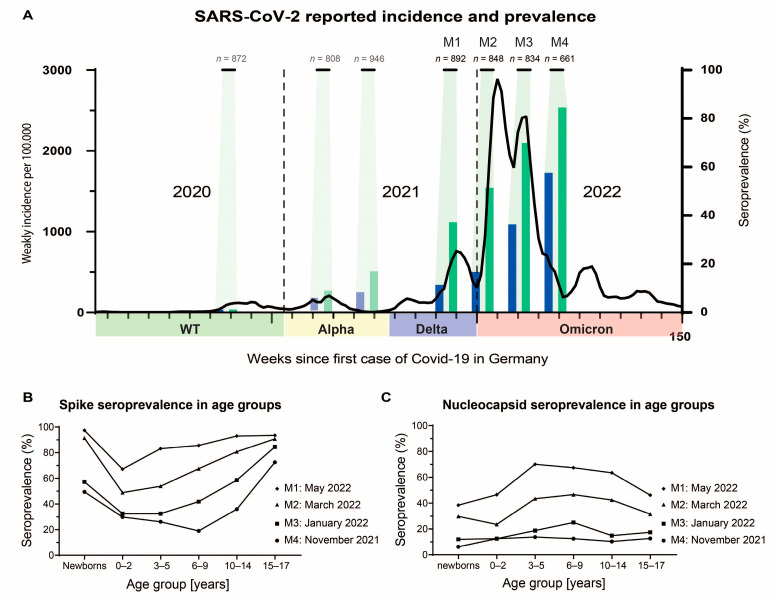
SARS-CoV-2 spike and nucleocapsid seroprevalence. (**A**) Comparison of the weekly COVID-19 incidence (black line) reported by the Robert Koch Institute in Germany with measured nucleocapsid (blue) and spike (green) seroprevalence. The dominant variant during the pandemic is displayed as follows: WT = wild type, green; Alpha = yellow; Delta = blue; Omicron = red. The time periods (M1–M4) for sample collections are marked in green with the respective number of included samples. The previous sample periods with their respective seroprevalences are shaded to aid interpretation [8]. (**B**) Spike protein seroprevalence for each age group and the respective sample period. (**C**) Nucleocapsid protein seroprevalence for each age group and the respective sample period.

In the following four sample periods before and after the new Delta and Omicron SARS-CoV-2 variants, a total of 4321 blood specimens were collected. A total of 330 samples were excluded from analyses for age (280), missing data (1), or insufficient material (49) and 756 samples only for seroprevalence and antibody level analyses due to multiple testing. Finally, of the 3235 samples, 892 were collected during period 1 (November 2021), 848 during period 2 (January 2022), 834 during period 3 (March 2022), and 661 during period 4 (May 2022) (Figure 1A). The proportion of female and male samples was balanced with a median (IQR) age of 7.88 (2.7–13.5); details are described in Table 1.

### 3.2. Seroprevalence Monitoring

During the four sample periods, overall spike seroprevalence increased from 37.56% to 51.89% to 70.50%, and it reached 85.17% in May 2022. The nucleocapsid seroprevalence increased from 11.55% to 16.98% to 36.69% and finally to 58.09%. The increase was statistically significant (*p* < 0.05), respectively. The development of overall spike and nucleocapsid seroprevalence is embedded in the course of the pandemic, with the cumulative incidence of PCR confirmed SARS-CoV-2 infections in children in Germany reported by the Robert Koch Institute (Figure 1A) [14]. Stratified by age, the seroprevalence differed significantly. Spike antibodies increased from the youngest up to the adolescents except the newborns. The latter showed a similar seroprevalence as the adolescents. Regarding all sample periods, similar trends were observed (Figure 1B). The nucleocapsid seroprevalence was significantly lower in newborns and children aged 0–2 and 15–17 compared to children aged 2–5 or 6–9 years. This effect became more prominent in the last two sample periods (all *p* < 0.05) (Figure 1C, Table 2). When comparing spike and nucleocapsid seroprevalence between sexes, no statistically significant difference between groups was found in all sample periods (Table 2). 

### 3.3. Antibody Levels Monitoring

**Figure 2:**
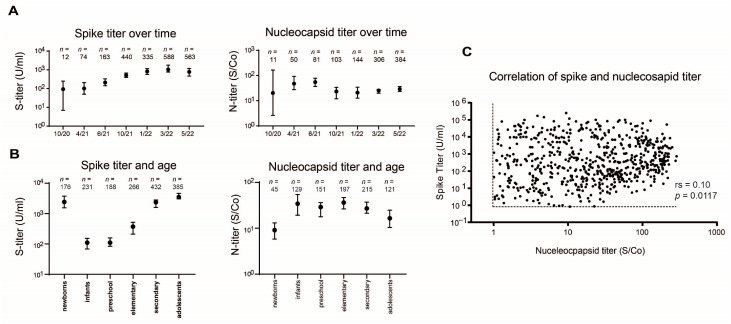
Spike and nucleocapsid antibody levels over time and age groups. (**A**) Spike and nucleocapsid antibody titers over time of the sample periods. The data of the first three time periods were published previously and are included for easier comparison [8]. Data are given as median with 95%CI. (**B**) Spike and nucleocapsid antibody titers according to the age group in all sample periods. Data are given as median with 95%CI. (**C**) Correlation of spike and nucleocapsid antibody levels. The black dotted lines represent the cutoff value used for each antibody assay. rs = Spearman correlation coefficient.

In addition, we conducted an analysis of immunity development in the pediatric population. Herein, a steady increase in the spike titer was seen in pediatric patients, with a maximum in March 2022 and a slight decrease thereafter. In contrast, the nucleocapsid titer was similar between all time periods (all *p* > 0.05) (Figure 2A). When considering the titers separately according to age groups, the spike titer showed an age-dependent increase from infancy onwards. Again, the nucleocapsid titer was significantly lower in newborns (*p* = 0.0002) and adolescents (*p* = 0.0097) but did not differ between other age groups (all *p* > 0.05) (Figure 2B). In the sample periods, no strong correlation was found between level of spike and nucleocapsid antibodies (Figure 2C).

### 3.4. Seroprevalence According to Diagnostic Groups

**Figure 3:**
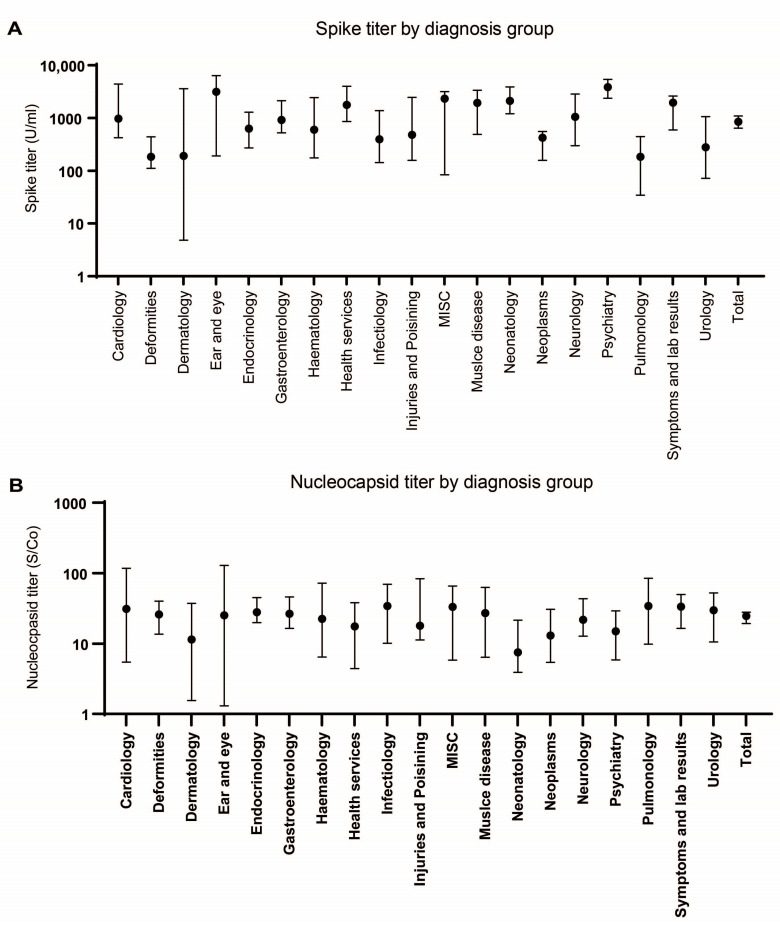
Spike and nucleocapsid antibody titers by diagnosis. (**A**) Spike and (**B**) nucleocapsid antibody titers by diagnosis group in all sample periods. Data are given as median with 95%CI.

For *n* = 2434 of the included samples, clinical data were available with *n* = 228 excluded due to duplication. First, samples were categorized based on the disease of the patients according to the chapters of the WHO recommended International Classification of Diseases (ICD-10). Secondly, all groups were analyzed for seroprevalence (Table 3) and antibody titer (Figure 3), accordingly. Spike seroprevalence was similar between all groups except for a 24.1% higher spike seroprevalence in children with neoplasms compared to the corresponding age group. No statistically significant difference in nucleocapsid seroprevalence among the various diagnosis groups was found (Table 3).

Regarding the antibody level, compared to the median total antibody level, significantly higher spike antibody levels were found in patients with a diagnosis from psychiatry (median 3809 U/mL vs. 847 U/mL, *p* = 0.0024) and significantly lower levels in patients with deformations (median 184 U/mL vs. 3809 U/mL *p* < 0.0001) and pulmonary disorders (median 184 U/mL vs. 3809 U/mL, *p* = 0.0090) (Figure 3A). Again, no differences were found for nucleocapsid antibodies between groups.

### 3.5. SARS-CoV-2 Antibody Decay

As samples were collected for all timepoints and then anonymized, for several participants, multiple samples were available for individual monitoring and consecutive calculation of the half-life time of the antibodies. Finally, we were able to calculate antibody half-life for 51 participants who displayed both nucleocapsid and spike antibody responses and for 167 participants who only had spike antibodies, presumably due to vaccination.

**Figure 4:**
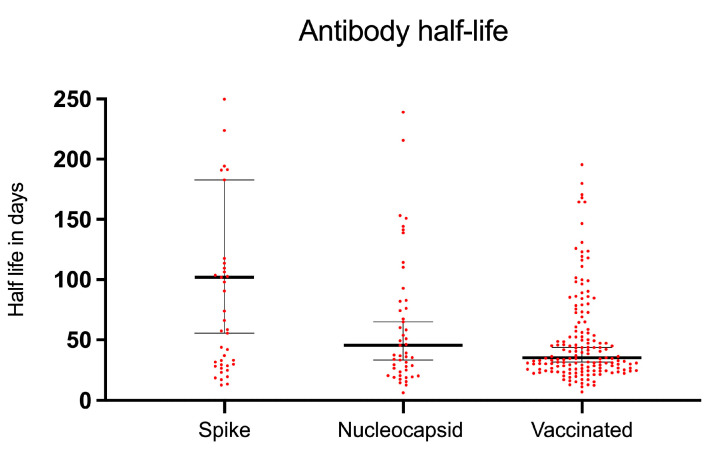
Antibody half-life compared between groups. Data are given as median with 95%CI. Spike = spike antibody half-life of patients with spike and nucleocapsid response. Nucleocapsid = nucleocapsid antibody half-life of patients with spike and nucleocapsid response. Vaccinated = spike antibody half-life of patients with spike but no nucleocapsid response who are presumably vaccinated.

For the first group, the median spike antibody half-life was 102 days (IQR 32 to 316), ranging from 13 to 2056 days. The median nucleocapsid antibody half-life was 45 days (IQR 25 to 93), ranging from 6 to 434 days. In the vaccinated group, the median spike antibody half-life was 35 days (IQR 26 to 69), ranging from 7 to 1807 days. The median antibody half-lives and the corresponding 95% confidence interval are displayed in Figure 4. Although the nucleocapsid half-life did not significantly differ compared to the spike half-life of vaccinated participants (*p* > 0.99), the spike antibody half-life was significantly longer compared to nucleocapsid antibody half-life and compared to the spike half-life in the vaccinated group (*p* = 0.0004) and to the nucleocapsid half-life (*p* = 0.0498). Furthermore, a correlation between spike and nucleocapsid half-life was found (r = 0.4060, *p* = 0.0031). But no significant correlation between initial antibody titer and half-life for both spike and nucleocapsid antibodies was found. We then calculated the number of days after which each participant would become seronegative using the initial antibody level and the corresponding antibody half-life. For the spike protein, our participants would turn seronegative after a median of 844 days compared to 208 days for the nucleocapsid protein and 292 for the vaccinated group. 

## 4. Discussion

In this study, we demonstrate the power of routine laboratory SARS-CoV-2 tests for long-term seroprevalence and humoral response monitoring in children. By using anonymized residual blood samples and routine clinical data, we were able to monitor general immunity and disease-specific immunity as well as the kinetics of SARS-CoV-2 antibody decay.

During our study, seroprevalence monitoring revealed a steady increase in nucleocapsid antibodies from 11.6% to 58.1% after the variant Omicron emerged. In line with this, spike antibody seroprevalence increased from 37.6% to 85.2%. These measures were in accordance with other studies reporting nucleocapsid antibody prevalence of 60.3% [17] and spike antibody seroprevalence studies of 69.5% [18] in May 2022 and 69.6% in July 2022 [17]. In our study, the highest prevalence of nucleocapsid antibodies as a marker of an infection was seen in the preschoolers aged 3–5 years and the lowest in toddlers from 0 to 2 years and adolescents from 15 to 17 years. In contrast, but in accordance with the anticipated vaccinations in older subjects, spike antibodies were lowest in toddlers and preschoolers and highest in adolescents. A recent worldwide systematic meta-analysis found a higher seroprevalence in older children compared to younger children as well [19]. Overall, our seroprevalence findings suggest a broad and widespread general immunity from either infection or vaccination.

In contrast to nucleocapsid antibody titers, the titers of spike antibodies exhibited a progressive increase during the course of the pandemic. This observed trend can be linked to the gradual escalation of vaccinations among adolescents, as it was not observed among children under the age of 12, who were not recommended for vaccination according to national guidelines. Given that spike antibodies are recognized as more pivotal and efficacious for conferring immunity in comparison to nucleocapsid antibodies, the observed augmentation in spike antibody levels alongside a diminished nucleocapsid response signifies that not only is immunity becoming more widespread (indicating an escalating seroprevalence) in the pediatric population but the quality of immunity is also notably improving over time due to a combination of vaccinations and infections. This development holds significant implications for the future impact of COVID-19 on healthcare systems. Notably, we observed lower nucleocapsid titers in newborns and adolescents. This observation may be a consequence of the high diaplacental transfer and vaccination rates in these groups, as vaccinated individuals tend to elicit a rapid and robust immune response, leading to a comparatively weaker nucleocapsid antibody response. Correspondingly, spike antibody levels were found to be highest among adolescents and newborns. In our tertiary-care center with patients of all pediatric specialties, no groundbreaking differences in nucleocapsid seroprevalence or nucleocapsid antibody titers between diagnosis groups were found. This could support a general validity of our above-described findings. The only significant deviation from the diagnosis groups to the total population was a higher spike seroprevalence in children with neoplasms, higher spike titers in psychiatry patients, and lower titers in patients of the deformations and of the pulmonary disorders groups.

Next, we demonstrated that by using multiple serial routine samples, the half-life of SARS-CoV-2 antibodies can be calculated. While the median half-life of spike antibodies was 102 days, the half-life of nucleocapsid antibodies was 45 days in patients with anticipated SARS-CoV-2 infection. Interestingly, in patients with spike antibodies only, the half-life was only 35 days. Similar antibody kinetics were found in other pediatric [14,15,16] and adult cohorts [20], too.

In conclusion, elimination of nucleocapsid antibodies (below cutoff) is approximately four times faster, fostering the idea of lower immune response to nucleocapsid protein in children [8]. The noteworthy disparity in the antibody half-life of spike antibodies between infected and presumably vaccinated children was previously observed [21]. These observations could be explained by the establishment of a robust hybrid immunity, integrating both vaccination and natural infection responses [22]. Consequently, this phenomenon appears to result in a decelerated decline in antibodies. These findings carry significant implications for future considerations regarding the necessity of booster shots.

Furthermore, we identified a significant variation of antibody half-life among our patients from a few days to hundreds of days. However, when spike antibodies wane quickly, nucleocapsid antibodies perform similarly, and vice versa. And as no significant correlation between antibody titer and antibody half-life was found, we hypothesize that the decay of antibodies remains consistent over time, too [23,24].

Our study possesses several limitations. Foremost, no information regarding infection and vaccination status was available according to the study design. However, the easy and broad applicability of routine laboratory SARS-CoV-2 assessment might allow general conclusions to be drawn. As a consequence, our data represent an estimation of the immunity in the pediatric population. Acknowledging that our data originate from patients, it is necessary to recognize that various health factors can potentially influence antibody levels and seroprevalence. The lack of clinical data regarding symptoms and disease severity might affect the diagnostic conclusiveness.

In summary, we demonstrate that anonymized routine laboratory SARS-CoV-2 tests allow long-term seroprevalence and humoral response monitoring in children with no burden to the patient and with cost-effectiveness for the healthcare system.

## Figures and Tables

**Table 1:** Patient characteristics of four time periods.

	All Periods	M1	M2	M3	M4
Samples (*n*)	3235	892	848	834	661
Male (%)	1683 (52%)	466 (52%)	453 (52%)	433 (52%)	331 (50%)
Female (%)	1552 (48%)	426 (48%)	395 (47%)	401 (48%)	330 (50%)
Age in years mean (SD ^1^)	8.12 (5.79)	7.91 (5.79)	8.24 (5.88)	8.32 (5.82)	7.96 (5.62)
Age in years median (IQR ^2^)	7.88 (2.7–13.5)	7.72 (2.4–13.0)	8.29 (2.6–13.7)	8.09 (2.8–13.8)	7.55 (2.8–13.1)

^1^ Standard deviation, ^2^ interquartile range.

**Table 2:** Seroprevalence of all time periods and ages. *n* = number of samples, S+ = spike reactive, N+ = nucleocapsid reactive.

Sample Period	Groups	Total *n*	S+ *n*	N+ *n*	Prevalence S+	*p*-Value *	Prevalence N+	*p*-Value *
Total	All	3235	1926	937	59.54%		28.96%	
	Male	1683	978	504	58.11% ^1^	0.0920 ^1^	29.95%	0.2008
	Female	1552	948	433	61.08% ^1^		27.90%	
	Newborns	263	176	45	66.92% ^4^	0.0106 ^4^	17.11%	0.0001
	0–2	595	261	138	43.87% ^4^	0.0001	23.19%	0.0005
	3–5	477	225	166	47.17% ^4^	0.0001	34.80%	0.0026
	6–9	596	307	216	51.51% ^4^	0.0001	36.24%	0.0001
	10–14	768	503	237	65.49% ^4^	0.0001	30.86%	0.1868
	15–17	536	454	135	84.70% ^4^	0.0001	25.19%	0.0370
Nov. 2021	All	892	335	103	37.56% ^2^	0.0001 ^2^	11.55%	0.0012
	Male	466	173	62	37.12%	0.7824	13.30%	0.0937
	Female	426	162	41	38.03%		9.62%	
	Newborns	99	49	6	49.49%	0.0112	6.06%	0.0931
	0–2	142	42	18	29.58%	0.0374	12.68%	0.6675
	3–5	138	36	19	26.09%	0.0022	13.77%	0.3849
	6–9	168	32	21	19.05%	0.0001	12.50%	0.6878
	10–14	203	73	21	35.96%	0.6214	10.34%	0.6178
	15–17	142	103	18	72.54%	0.0001	12.68%	0.6675
Jan. 2022	All	848	440	144	51.89% ^2,3^	0.0001 ^3^	16.98%	0.0001
	Male	453	222	86	49.01%	0.0738	18.98%	0.0997
	Female	395	218	58	55.19%		14.68%	
	Newborns	75	43	9	57.33%	0.3355	12.00%	0.2624
	0–2	151	49	19	32.45%	0.0001	12.58%	0.1211
	3–5	117	38	22	32.48%	0.0001	18.80%	0.5958
	6–9	148	62	37	41.89%	0.0086	25.00%	0.0056
	10–14	208	122	31	58.65%	0.0255	14.90%	0.3960
	15–17	149	126	26	84.56%	0.0001	17.45%	0.9044
Mar. 2022	All	834	588	306	70.50% ^3^	0.0001	36.69%	0.0001
	Male	433	308	167	71.13%	0.7043	38.57%	0.2506
	Female	401	280	139	69.83%		34.66%	
	Newborns	48	44	14	91.67%	0.0005	29.17%	0.2848
	0–2	173	84	41	48.55%	0.0001	23.70%	0.0001
	3–5	115	62	50	53.91%	0.0001	43.48%	0.1180
	6–9	148	100	69	67.57%	0.4266	46.62%	0.0064
	10–14	198	160	84	80.81%	0.0002	42.42%	0.0631
	15–17	152	138	48	90.79%	0.0001	31.58%	0.1631
May 2022	All	661	563	384	85.17%		58.09%	
	Male	331	275	189	83.08%	0.1546	57.10%	0.6364
	Female	330	288	195	87.27%		59.09%	
	Newborns	41	40	16	97.56%	0.0205	39.02%	0.0137
	0–2	129	86	60	66.67%	0.0001	46.51%	0.0038
	3–5	107	89	75	83.18%	0.5523	70.09%	0.0073
	6–9	132	113	89	85.61%	1.00000	67.42%	0.0178
	10–14	159	148	101	93.08%	0.0008	63.52%	0.1177
	15–17	93	87	43	93.55%	0.01160	46.24%	0.0169

* Fisher’s exact test was used for the comparison of all seroprevalence data. A gender-based comparison of seroprevalence was conducted for each sample period (e.g., superscript number ^1^). The overall seroprevalence for each sample period was compared to the subsequent sample period, respectively (e.g., superscript numbers ^2,3^). Within each sample period, the seroprevalence of each age group was compared with the seroprevalence of the remaining age groups (e.g., superscript number ^4^). *p* < 0.05 indicates statistical significance.

**Table 3:** Seroprevalence by diagnosis groups. S = spike, N = nucleocapsid, *n* = number of samples, MISC = multisystem inflammatory syndrome in children.

Diagnosis Group	Prevalence S	*p*-Value *	Prevalence N	*p*-Value *	*n*	Median Age
Cardiology	64.7%	0.8609	31.4%	0.7295	51	12.3
Deformities	56.4%	0.4160	27.5%	0.5059	273	2.0
Dermatology	42.9%	0.4101	32.1%	0.5266	28	5.9
Ear and eye	56.5%	0.3466	26.1%	0.4823	23	11.8
Endocrinology	59.3%	0.4058	36.9%	0.9156	290	9.2
Gastroenterology	68.8%	0.8039	33.0%	0.8068	176	11.6
Hematology	55.8%	0.2244	28.3%	0.5083	120	7.2
Health services	61.6%	0.3857	14.1%	0.5283	99	0.0
Infectiology	52.9%	1.000	34.5%	0.4043	87	5.8
Injuries/poisoning	59.7%	0.3847	34.7%	0.4609	72	6.0
MISC	100.0%	0.2482	100.0%	0.0657	3	5.3
Muscle disease	67.6%	0.2840	28.4%	0.8784	74	14.1
Neonatology	70.3%	0.5067	17.2%	1.000	145	0.0
Neoplasms	74.8%	0.0001	42.1%	0.2498	107	7.6
Neurology	58.8%	0.5274	38.6%	0.8973	114	9.1
Psychiatry	78.4%	0.4834	33.3%	0.2934	111	14.4
Pulmonology	39.7%	0.2297	24.4%	0.1871	131	2.7
Symptoms/lab results	58.9%	0.0682	26.0%	0.7186	192	11.3
Urology	54.5%	0.5379	29.1%	0.3556	110	8.4
Total	60.6%	0.0035	30.1%	0.5229	2206	7.1

* Fisher’s exact test was used for the comparison of all seroprevalence data. The seroprevalence status of each diagnosis group was compared to the seroprevalence in the corresponding age group. *p* < 0.05 indicates statistical significance.

## Data Availability

Data are contained within the article.
